# Integrated Ozonation Ni-NiO/Carbon/g-C_3_N_4_ Nanocomposite-Mediated Catalytic Decomposition of Organic Contaminants in Wastewater under Visible Light

**DOI:** 10.3390/nano14020190

**Published:** 2024-01-14

**Authors:** Abdullah Y. Alhato, Rajeev Kumar, Mohammad A. Barakat

**Affiliations:** Department of Environment, Faculty of Environmental Sciences, King Abdulaziz University, Jeddah 21589, Saudi Arabia; a.y.alhato2013@gmail.com (A.Y.A.); mabarakat@gmail.com (M.A.B.)

**Keywords:** hybrid catalytic process, photocatalysis, ozonation, wastewater treatment

## Abstract

Developing a hybrid process for wastewater purification is of utmost importance to make conventional methods more efficient and faster. Herein, an effective visible light-active nickel–nickel oxide/carbon/graphitic carbon nitride (Ni-NiO/C/g-C_3_N_4_)-based nanocatalyst was developed. A hybrid process based on ozonation and Ni-NiO/C/g-C_3_N_4_ visible light photocatalysis was applied to decolourize the Congo red (CR), Alizarin Red S (ARS), and real dairy industry wastewater. The synthesized catalyst was characterized using scanning electron microscopy (SEM), energy dispersive X-ray spectroscopy (EDX), Χ-ray powder diffraction (XRD), X-ray photoelectron spectroscopy (XPS), and UV-Vis diffuse reflectance spectrophotometry (UV-Vis DRS). The factors affecting the catalytic process were evaluated, including contact time, solution pH, initial dye concentration, etc. The degradation rate of CR and ARS was compared between the photocatalysis, ozonation, and integrated photocatalytic ozonation (PC/O_3_) methods. The results showed 100% degradation of CR and ARS within 5 min and 40 min, respectively, by integrated PC/O_3_. The reusability of the modified catalyst was evaluated, and four successive regenerations were achieved. The modified Ni-NiO/C/g-C_3_N_4_ composite could be considered an effective, fast, and reusable catalyst in an integrated PC/O_3_ process for the complete decolourization of wastewater.

## 1. Introduction

Water is an essential resource on the earth and vital to economic and industrial activities [1]. Natural, domestic, and industrial activities have caused an increase in pollutants in surface water [2]. The textile and food industries use many dyes and colours to colour their products. Polaris market research reported a $10.68 billion dyes market in 2021 and it is probable to grow at a compound annual growth rate of 4.7% up to 2030 [3]. A similar growth of about $4.77 billion is expected for food colours by 2026 [4]. The fast growth in the textile and food industries is expected to produce more coloured wastewater containing synthetic organic chemicals, which are hazardous to living systems [5]. The type and toxicity levels of pollutants depend on the effluents, compounds, and their concentration in the water [6,7]. Congo red (CR) and Alizarin Red S (ARS) dyes are used for different purposes and discharged into the environment [8,9]. The release of both dyes into water bodies can contaminate the water, leading to poor water quality, toxic impacts on aquatic organisms, and, ultimately, danger to human health and the ecosystem [10,11]. Therefore, it is essential to develop appropriate treatment methods for removing colouring chemicals from contaminated water to prevent their negative environmental impacts [12].

Various methods have been employed to reduce/decontaminate pollutants from wastewater, including advanced oxidation processes (AOPs), membrane separation, aerobic/anaerobic treatments, and adsorption. AOPs are one of the most effective options for completely decomposing and mineralizing organic contaminants [13]. AOPs like photocatalysis are an effective method for degrading organic species. However, the slow decomposition rate is an issue with photocatalysis. Integrating photocatalysis with other AOPs could be an effective approach to obtain better results in a short duration. The integration of ozonation with photocatalysis (PC/O_3_) could be an effective approach to the wastewater treatment process due to the strong oxidation potential of ozone (O_3_) [14]. It has been reported that photocatalytic ozonation produces a higher rate of ^•^OH radicals than other oxidation processes due to the more electrophilic nature of O_3_ towards photo-generated electrons than O_2_. This results in quicker decomposition of organics and mineralization through the PC/O_3_ process [14]. Previous studies explored the potential of integrated PC/O_3_ and suggested that it is highly effective and environmentally friendly [13,14,15]. However, Silva et al. [14] and Kang [15] used the UV light active photocatalytic, which makes the process costlier, and higher O_3_ is required due to its fast photolysis in the presence of UV light [13]. Therefore, selecting the photocatalyst for the PC/O_3_ process is an important aspect of making the process more economical.

Numerous semiconductor catalysts have been used for the photodegradation of organic pollutants such as tungsten oxide (WO_3_) [16], zinc oxide (ZnO) [17], titanium dioxide (TiO_2_) [18], carbon nitride (g-C_3_N_4_) [19], nickel oxide (NiO) [20], etc. NiO is a widely applied semiconducting catalyst with good chemical stability and catalytic properties [20]. NiO catalysts with wide band gaps have been used to degrade organic pollutants, but bare NiO was less efficient in the visible light region. Numerous strategies have been applied to alter the band gap energy and to make visible light-active catalysts, such as doping, co-doing, coupling, defect creation, hybrid composite formation, etc. Hybrid catalysts can increase efficacy, decrease the band gap, and improve the dispersion proficiency of excited electron–hole pairs. Moreover, pristine metal oxides sometimes face problems with the separation of charges and encounter the movement of redox reactions [21,22,23,24]. Synthesizing a hybrid catalyst with other semiconductors with narrow band gaps is desirable to overcome such restrictions [25,26]. Among others, hybrid materials-based carbon nanomaterials such as carbon, carbon nitride (g-C_3_N_4_), etc., with excited properties including good catalyst support, chemical stability, and nontoxicity, can be combined with NiO to improve the catalytic properties in the visible light region [27,28]. In addition, carbon and C_3_N_4_ can be fabricated with low-cost and naturally abundant precursors [29]. g-C_3_N_4,_ with a band gap of 2.8 eV, is a chemically stable and non-hazardous metal-free semiconductor photocatalyst, which is responsible for absorbing visible light regions, and it was suggested to be the best option to produce nano-heterostructures. Moreover, g-C_3_N_4_ can be easily synthesized by using economically available precursors such as melamine. Though NiO acts as a photocatalyst, the efficiency is low compared to g-C_3_N_4_. Coupling NiO with g-C_3_N_4_ could be a promising approach to developing a visible light-active catalyst. However, NiO and g-C_3_N_4_ could be photoexcited under light irradiation, and the results might be less promising. Therefore, another material that can separate the e^−^/h^+^ pair more effectively should be introduced. Forming a ternary composite of carbon, NiO, and g-C_3_N_4_ could be promising in obtaining a better visible light catalyst. Carbon is not a semiconductor but can separate the e^−^/h^+^ pair generated by the NiO and g-C_3_N_4_ under light irradiation.

Waste cellulosic tissue paper is a good source of nanocarbon. The distinct structure of tissue paper comprises an interwoven network of cellulose fibres, mainly composed of C and O components. Additionally, tissue paper has consistent qualities, including porosity, density, and composition, because it is an established industrial product [30]. Li et al. [31] modified a hierarchically porous Cu–Ni/C composite catalyst by direct calcination processes in a furnace at 700 °C for carbonization at a 5 °C/min heating rate. Microporous networks with a high surface area (538 m^2^/g) were created from interwoven carbon fibres loaded with nano Cu–Ni particles using tissue paper as a bio-template for template-directed synthesis. High catalytic activity was shown by the hierarchically porous structures that were produced. These findings have motivated us to fabricate hierarchically porous catalysts by using tissue paper as a carbon template. High catalytic activity, a large surface area, and an accessible mass transport pathway of a microporous network are the anticipated features of hierarchically porous Ni-NiO/C/g-C_3_N_4_ networks. Therefore, synthesizing a ternary composite of NiO with carbon (C) and C_3_N_4_ without additional chemical treatment could be an environmentally friendly way to develop a new catalyst [32]. Further, NiO on carbonaceous support could prevent the potential agglomeration of NiO and subsequently would gain conducive ground for the maximum utilization of catalytic properties ascending from the surface [33]. In addition, the C/g-C_3_N_4_ support can provide fast mobility of excited electron–hole pairs, lead to safety from NiO photo corrosion, confer better mass diffusion, and have several reusability potentials through the long-term sustainment of photostability [34,35].

In this study, a novel hybrid Ni-NiO/C/g-C_3_N_4_ catalyst was synthesized using the facile approach. The synergy between the Ni-NiO, carbon, and g-C_3_N_4_ enhances the total number of active sites and suppresses the recombination of the photo-generated electrons/holes. The Ni-NiO/C/g-C_3_N_4_ composite catalyst was fabricated and subsequently applied to degrade CR and ARS from wastewater in an integrated photocatalytic ozonation (PC/O_3_) process under visible light. The factors affecting the PC/O_3_ process were evaluated, including contact time, solution pH, initial dye concentrations, etc. The degradation rate of CR and ARS were compared between photocatalysis, ozonation, and integrated PC/O_3_ processes. The mechanism of the synergetic effect of photocatalysis and ozonation on pollutant degradation was investigated to understand the integrated PC/O_3_ process.

## 2. Materials and Methods

### 2.1. Chemicals

Nickel chloride hexahydrate (NiCl_2_·6H_2_O) and Congo red (CR) dye were purchased from Techno Pharmachem Hariyana (Haryana, India). The Alizarin Red S (ARS) and melamine were collected from the BHD Chemical, Poole, England. The used tissue papers were collected from the industrial waste management laboratory at King Abdulaziz University. The collected tissue papers were disinfected under UV light for 30 min, washed several times with hot deionized water, and dried at 105 °C for 15 h. 

### 2.2. Synthesis of Catalysts 

#### 2.2.1. Synthesis of g-C_3_N_4_

The graphitic carbon nitride (g-C_3_N_4_) was prepared by the direct calcination of melamine at 600 °C for 3 h, and the heating rate was 5 °C/min. The yellow powdered g-C_3_N_4_ was washed with deionized water and dried at 50 °C.

#### 2.2.2. Synthesis of NiO

The NiO was synthesized using nickel chloride hexahydrate (NiCl_2_·6H_2_O) salt. First, 2.0 g of NiCl_2_·6H_2_O was taken in a crucible and heated at 600 °C (5 °C/min) for 3 h. Thereafter, the obtained material was collected after cooling the oven and washed several times with DI water and ethanol. The filtered material was then dried at 105 °C for 14 h. 

#### 2.2.3. Synthesis of Ni-NiO/C/g-C_3_N_4_

Initially, 0.5 g of g-C_3_N_4_ was mixed in 50 mL of deionized water and stirred for 30 min. Thereafter, 3.1 g of NiCl_2_·6H_2_O and 4.5 g of tissue paper were added to the same solution and stirred continuously. After 15 min of stirring, the mixture was dried at 105 °C for 24 h. Next, the materials were calcined at 600 °C (5 °C/min) for 3 h. Then, the resultant Ni-NiO/C/g-C_3_N_4_ composite was washed several times with deionized water, acetone, and ethanol. Finally, the synthesized Ni-NiO/C/g-C_3_N_4_ composite was dried for 14 h at 105 °C.

#### 2.2.4. Characterization of Photocatalysts

The morphological features of materials and their elemental presence were characterized by scanning electron microscopy coupled with energy dispersive X-ray spectroscopy (EDX) using JSM-7500 F, JEOL, Tokyo, Japan. The crystal structure of the photocatalysts was analysed by Χ-ray powder diffraction (XRD) using a Rigaku Ultima IV XRD analyser from Tokyo, Japan. To investigate the band gap of photocatalysts, they were analysed using UV–visible diffuse reflectance spectroscopy (UV–vis-DRS). A2Z Ozone Inc., Louisville, KY, USA was used for the ozonation experiment.

#### 2.2.5. Photocatalytic Ozonation Studies 

Photocatalysis, ozonation, and integrated PC/O_3_ studies were conducted by mixing 0.05 g catalyst in 50 mL solution under 108 W visible light illumination and 20 g/h of ozone flow. The factors affecting the photocatalytic ozonation process, including solution pH, contact time, the concentration of dyes, etc., were evaluated. The effect of solution pH on the degradation process was evaluated in the pH range of 2 to 9. The initial CR and ARS concentration effect was investigated between 10 mg/L and 160 mg/L. The effect of contact times was evaluated to optimize the CR and ARS degradation rate under single and integrated PC/O_3_ processes to 0 to 180 min. The CR and ARS degradation rate was analysed using a UV–Vis DR 6000 spectrophotometer at an λmax of 495 nm and 423 nm, respectively.

## 3. Results and Discussion

### 3.1. Characterization of Catalysts

**Figure 1:**
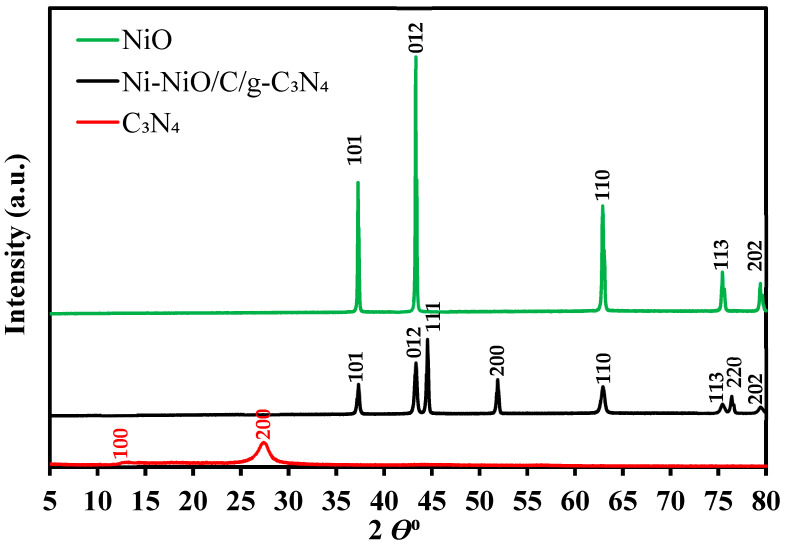
XRD pattern of the NiO, g-C_3_N_4_, and Ni-NiO/C/g-C_3_N_4_ hybrid nanocomposite.

The XRD patterns of the C_3_N_4_, NiO, and Ni-NiO/C/g-C_3_N_4_ composites are presented in Figure 1. The C_3_N_4_ crystal structure shows peaks around 13.04° (100) and 27.55° (200), which are also shown in the literature at almost the same region [36]. The diffraction peaks of NiO are indexing (PDF 01-089-7390) with representing planes at around 2θ° of 37.24 (101), 43.29 (012), 62.84 (110), 75.414 (113), and 79.384 (202). The diffraction peak of the Ni-NiO/C/g-C_3_N_4_ composite catalyst is presented in the XRD pattern at 2θ° with corresponding planes of 37.43, 43.33, 51.7, 63.11, 75.31, and 79.384 (202), and these records are well-matched with the NiO rhombohedral structure (PDF 01-089-7390). Although, new peaks at 2 θ°, 44.49 (111), 52.84 (200), and 76.37 (220) were observed in the XRD spectrum of Ni-NiO/C/g-C_3_N_4,_ which indicates the presence of Ni (PDF 01-071-3740). Ni may be formed due to the partial reduction of the Ni precursor during the calcination of the tissue paper, which produces CO and CO_2_ after burning. The presence of CO and CO_2_ hindered the oxidation of the nickel. Therefore, a mixed phase of Ni-NiO was formed due to less oxygen during calcination [37]. However, the carbon and g-C_3_N_4_ peaks were not observed due to their small amount and low peak intensity compared to NiO in the Ni-NiO/C/g-C_3_N_4_ nanohybrid [38].

**Figure 2:**
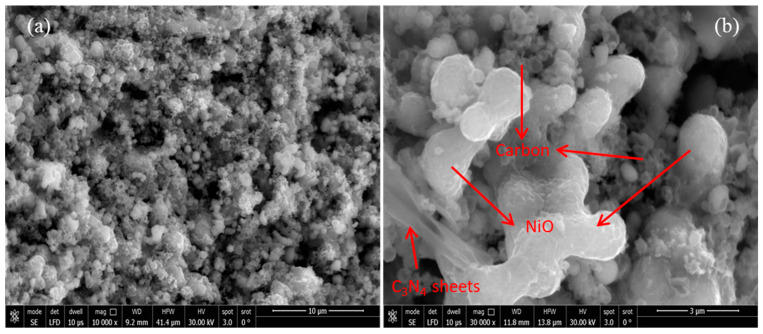
SEM images of the Ni-NiO/C/g-C_3_N_4_ composite at different magnifications: (**a**) 10k, (**b**) 30k.

The morphological features and surface texture of the NiO/C/g-C_3_N_4_ catalyst were analysed by a scanning electron microscope (SEM) as depicted in Figure 2. The SEM images are shown in Figure 2a showing the fine particle structures. Figure 2b exhibits the spherical shape of the NiO, and some of the irregular, wrinkled sheet-like attachments appear due to the incorporation of carbon and g-C_3_N_4_ with NiO. The spherical agglomerate shape from NiO was reported in the previous study [39], and the stacked sheet-like morphology was defined for g-C_3_N_4_ [36]. Moreover, the EDX profile of the NiO/C/g-C_3_N_4_ composite confirmed the following chemical elements with their atomic percentage states as Ni (49.12%), C (22.26%), O (22.62%), and N (1.01%). 

**Figure 3:**
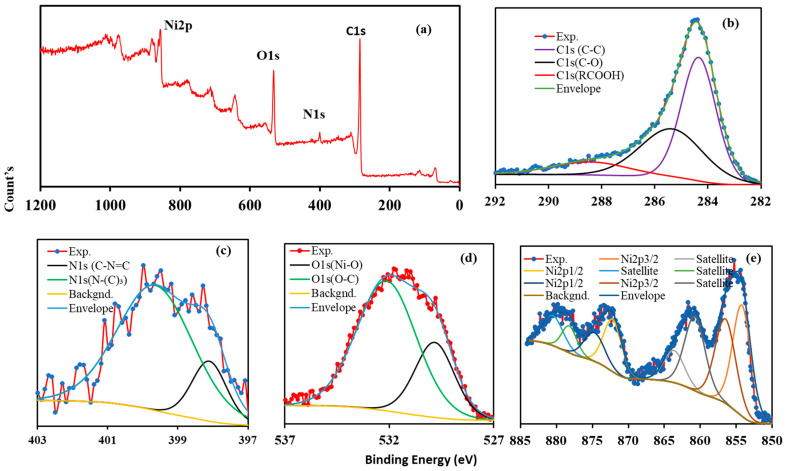
XPS of Ni-NiO/C/g-C_3_N_4_ composite catalyst: (**a**) wide scan survey, (**b**) C1s, (**c**) N1s, (**d**) O1s, (**e**) Ni2p.

The chemical composition of the NiO/C/g-C_3_N_4_ catalyst was recorded using XPS analysis as illustrated in Figure 3. The complete scan survey of the composite catalyst is exhibited in Figure 3a, indicating the presence of C1s (285.42 eV), N1s (399.93 eV), O1s (532.21 eV), and Ni2p_3/2_ (856.24 eV). Figure 3b represents the wide scan C1s spectra deconvoluted into 284.34, 285.36, and 288.43 (eV), representing the sp^2^ (C–C bonds), C-O, and sp^2^-bonded carbon in (RCOOH). These peaks for C1s agree with the reported literature [36]. Figure 3c shows two spectra for N1s deconvoluted into 393.1.88 and 399.64 eV. The O1s spectra in Figure 3d with one high-intensity bump at around 529.83 eV and low-intensity peak at 532.13 eV confirm the N-O bond and O^2-^ oxidation state, respectively [40]. Figure 3e exhibits several peaks with corresponding binding energy at 854.03, 863.63, and 872.18, belonging to the Ni2p_3/2_, while the other peaks appearing at 880.48, 878.21, 874.7, 856.01, and 860.76 eV represent the Ni2p_1/2_ and satellite peaks. However, the XPS peak for metallic Ni generally appeared around 852.6 eV and was not observed in the deconvoluted spectra of Ni2p_3_. Generally, metallic Ni in the NiO lattice structure and XPS scan the surface of the materials, which typically do not appear in the XPS spectrum. Similar results were also reported in previously reported articles [41,42]. The recorded chemical elements of C1s, O1s, N1s, and Ni2p_3/2_ in the composite catalyst with their peaks, spectra, and binding energy confirm the successful formation of the NiO/C/g-C_3_N_4_ hybrid catalyst. 

### 3.2. Optical Characterization of Catalysts

**Figure 4:**
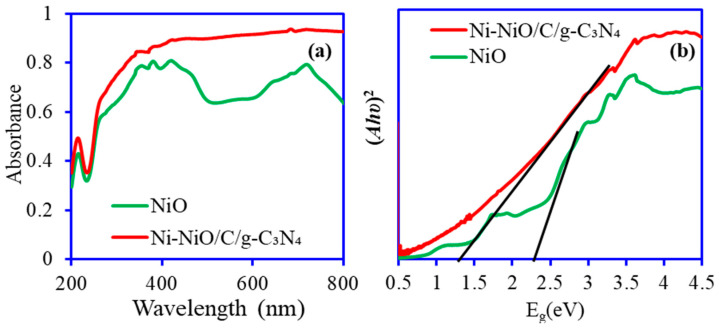
(**a**) UV-visible absorption spectrum. (**b**) Tauc plots of NiO and Ni-NiO/C/g-C_3_N_4_ nanohybrid.

The light absorption properties and band gap analysis of NiO and the Ni-NiO/C/g-C_3_N_4_ nanohybrid are depicted in Figure 4. The UV-visible spectra of NiO and Ni-NiO/C/g-C_3_N_4_ are depicted in Figure 4a. Figure 4a displays an absorption edge around 224 nm, which is attributed to the electronic transitions from the ionized oxygen vacancies (IOV) to the valence band (VB) of the NiO. These transitions may arise due to existing defects in NiO nanostructures [43]. The band gap energy (E_g_) of the NiO and Ni-NiO/C/g-C_3_N_4_ nanohybrid was calculated using the equation (*Ahυ*)*^n^* = *B*(*hυ* − *E*_g_) (where: *hυ*—photo energy; *A*—absorbance; *B*—material constant; and *n* is 2 or 1/2 for direct and indirect transitions). Figure 4b illustrates the band gap for pure NiO and Ni-NiO/C/g-C_3_N_4_ as 2.36 eV and 1.45 eV, respectively. NiO band gap energy is generally above 3.2, but thermally synthesized NiO materials show a lower E_g_ 2.83 eV [43]. Moreover, Hosny [44] reported that the different precursors also have a role in directing the E_g_ of the NiO and observed NiO nanoparticles’ E_g_: 3.12, 2.93, 2.82, and 2.45 eV using different precursors calcinated at 700 °C. The optical analysis revealed that NiO and Ni-NiO/C/g-C_3_N_4_ are capable of absorbing visible light.

### 3.3. Degradation of CR and ARS

#### 3.3.1. Photocatalytic and Ozonation Studies

**Figure 5:**
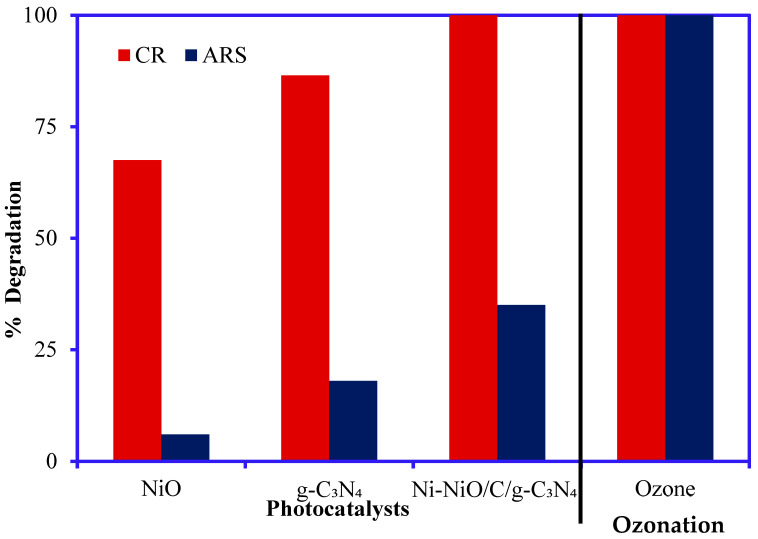
Comparison of the photocatalytic performance of NiO, g-C_3_N_4_, and Ni-NiO/C/g-C_3_N_4_ composite and the ozonation catalytic degradation of CR and ARS (concentration 20 mg/L, pH 5.0 for CR and 9.0 for ARS, contact time 180 min, Vol. 50 mL, catalyst mass 0.05 g).

The photocatalysis and ozonation degradation studies were conducted to compare and optimize the CR and ARS degradation. The degradation rate of CR and ARS was compared between photocatalysis and ozonation. The photocatalytic performance of the Ni-NiO/C/g-C_3_N_4_ catalyst was compared with pure NiO, and g-C_3_N_4_ for degrading CR and ARS under visible light illumination. The degradation results of CR and ARS are presented in Figure 5. The results showed that the composite catalyst had the highest degradation rate for CR as complete mineralization (100%). In comparison, 40% degradation was achieved for ARS. The trinary composite had higher degradation than the pure catalysts because of the synergetic effect, and other catalysts may act as interfacial charge transporters in the composite catalyst. The interfacial charge activities prevented electron–hole (e^−^/h^+^) pair recombination, subsequently enhancing irradiation performance [45,46] because of the improved photocatalytic properties and the subsequent highest degradation rate of CR and ARS.

The degradation of CR and ARS was also tested under the same experimental conditions using ozone (without light and catalysts), and the results are depicted in Figure 5. The primary results demonstrate that the ozonation can decompose the CR and ARS completely. Therefore, the Ni-NiO/C/g-C_3_N_4_ composite was considered a catalyst for the initial photocatalytic ozonation studies. The complete degradation of CR and ARS by ozone occurs through direct oxidation by ozone molecules and indirect oxidation by ^•^OH and ^•^O_2_^−^ [47]. Based on these primary results, the degradation of CR and ARS under different experimental conditions was conducted to find the optimum decomposition in the single and integrated catalysis processes.

#### 3.3.2. Effect of pH

**Figure 6:**
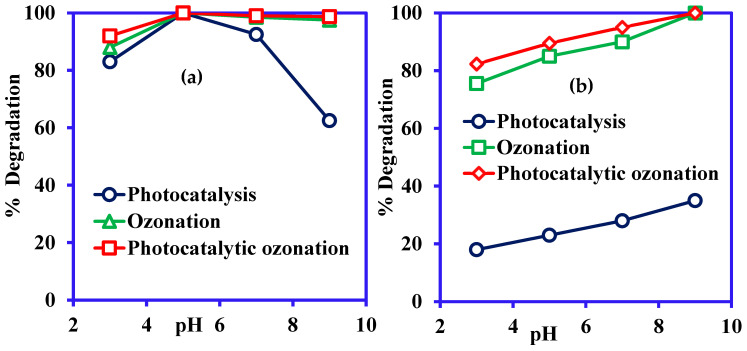
Effect of pH on photocatalysis, ozonation, and integrated PC/O_3_: (**a**) CR degradation, (**b**) ARS degradation (concentration 20 mg/L; contact time 180 min; vol. 50 mL; catalyst Ni-NiO/C/g-C_3_N_4_; catalyst mass 0.05 g; ozone flow rate 20 g/h).

The effect of solution pH was investigated to optimize the best degradation rate of CR and ARS in photocatalysis (PC), ozonation (O_3_), and integrated PC/O_3_ process within the range of pH 3.0 to 9.0. The results are depicted in Figure 6a and Figure 6b for CR and ARS degradation, respectively. The experimental results showed that the best CR degradation efficiencies were found in the acidic condition at pH 5.0 and that 100% CR degradation was recorded. The degradation percentages decreased with the increment of solution pH. This can be explained based on the surface interaction between the Ni-NiO/C/g-C_3_N_4_ nanohybrid and CR dye molecules, which interact due to electrostatic forces on the catalyst’s surface. In the acidic solution, H^+^ was adsorbed on the surface of Ni-NiO/C/g-C_3_N_4_ nanohybrid, generating a positive charge on its surface and attracting the anionic CR molecules. The basic medium showed lower catalysis due to electrostatic repulsion between anionic CR molecules and the net negative charge generated by the ^−^OH ions on the surface of Ni-NiO/C/g-C_3_N_4_ [48]. The better interaction between dye molecules and Ni-NiO/C/g-C_3_N_4_ favours their decomposition. On the other hand, the opposite behaviour was shown for the photocatalytic degradation of ARS as presented in Figure 6b, and the highest degradation (40%) of ARS was in the alkaline condition at pH 9.0. The opposite charges of the ARS and Ni-NiO/C/g-C_3_N_4_ attracted each other and showed the best results in the basic medium due to the cationic ARS and anionic Ni-NiO/C/g-C_3_N_4_ interactions [49].

In the ozonation process, the best efficiency was found in the alkaline condition compared to the acidic condition for ARS. For CR degradation, the best efficiency was found in acidic media compared to alkaline conditions. This may be due to the ozone depletion reaction. When pH increases, it will affect the ozone movement from a gas to a liquid phase in both forward and backward oxidation pathways, as reported in previous work [50]. In addition, a high rate of ^•^OH in the alkaline condition is more effective in oxidation than abundant ozone for ARS. Therefore, the alkaline conditions influenced the indirect oxidation and subsequently enhanced the ARS removal capacity compared to the acidic medium.

The integrated PC/O_3_ process showed that the degradation of the CR and ARS increases with the rise in solution pH. The integrated PC/O_3_ process produces higher ^•^OH than photocatalysis or ozonation, which showed the enhanced decomposition of the CR and ARS molecules. Moreover, higher ^•^OH radicals are produced in the basic medium due to the ^−^OH ions. Therefore, a better decomposition of the dye molecules was observed in the basic medium [51].

#### 3.3.3. Effect of Contact Time

**Figure 7:**
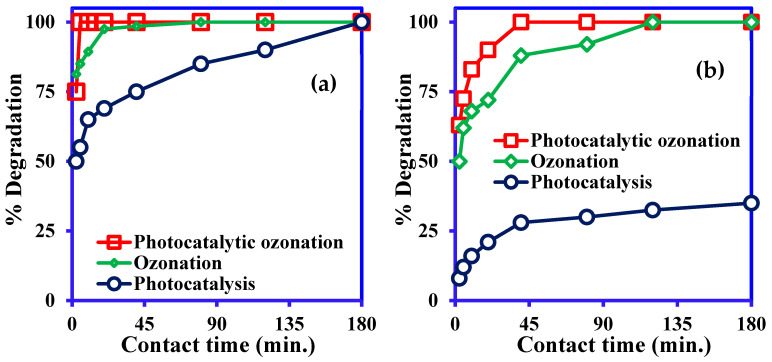
Effect of contact time on photocatalysis, ozonation and integrated photocatalytic ozonation: (**a**) CR degradation, (**b**) ARS degradation (concentration 20 mg/L for both dyes, pH 5.0 for CR and 9.0 for ARS, Vol. 50 mL, catalyst: Ni-NiO/C/g-C_3_N_4_, catalyst mass 0.05 g, ozone flow rate 20 g/h).

The effect of contact times was also evaluated for the optimization of degradation time CR and ARS in PC, O_3_, and integrated PC/O_3_, and the results are presented in Figure 7, respectively. The results exhibited that the completed degradation of CR occurred within 180 min, while only 40% mineralization of ARS was achieved by Ni-NiO/C/g-C_3_N_4_ photocatalysis. On the other hand, O_3_ showed 100% degradation of CR and ARS within 90 and 120 min, respectively. The integrated PC/O_3_ process demonstrated 100% CR removal within 5 min and ARS completely degraded within 40 min of reaction time. This happened because of the synergistic actions of PC and O_3_, which produce strong reactive radicals that decompose the dye molecules faster [13,52].

#### 3.3.4. Effect of Initial Concentration

**Figure 8:**
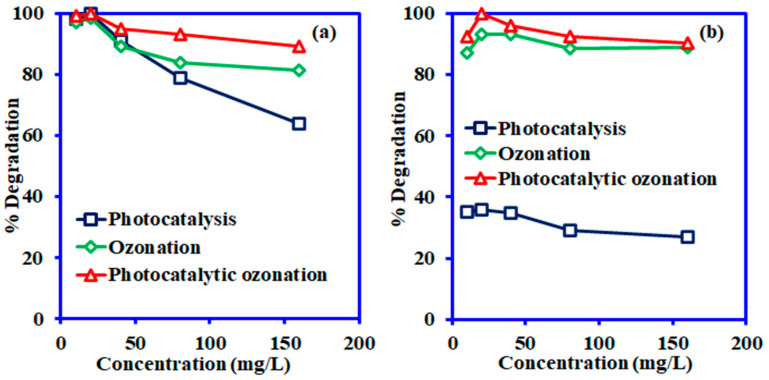
Effect of initial concentration of photocatalysis, ozonation, and integrated photocatalytic ozonation: (**a**) CR degradation, (**b**) ARS degradation (pH 5.0 for CR and 9.0 for ARS, contact time 180 min, Vol. 50 mL, catalyst: Ni-NiO/C/g-C_3_N_4_, catalyst mass 0.05 g, ozone flow rate 20 g/h).

The effect of the initial concentrations of CR and ARS was investigated from 10 mg/L to 160 mg/L at optimized pH 5.0 for CR and 9.0 for ARS and the results are illustrated in Figure 8. The percentage of the degradation of CR and ARS initially at low concentrations was high for both dyes. When the initial concentrations of the dyes were increased, the degradation performance was also slower than at low concentrations, and 20 mg/L of both dye concentrations reached optimum degradation in all three degradation methods. The photocatalytic activity by the Ni-NiO/C/g-C_3_N_4_ composite was comparatively weakened at higher dye concentrations due to an increase in the viscosity and subsequent decline of optical density, which hindered the penetration of the photons into the sample and consequently gradually declined the photoexcitation of electrons [49]. On the other hand, in the ozonation process, the percentages of CR and ARS degradations initially were also high, and while increasing the initial concentrations, CR and ARS subsequently molecules increased in the solution, but the competition of ^•^OH radicals and the shortage of ^•^OH production from O_3_ depletion was influenced the CR and ARS degradation capacity. The same behaviour was also reported in a previous study [51].

#### 3.3.5. Comparison of Efficiency with Literature

Combined photocatalysis and ozonation processes can eliminate organic pollutants more efficiently because the combined process generates more ROS. A table for the previously reported studies compared with the Ni-NiO/C/g-C_3_N_4_ catalyst used in this study is tabulated in Table 1. As seen, the photocatalytic ozonation in the presence of Ni-NiO/C/g-C_3_N_4_ catalyst was greater for CR and ARS degradation. It has been reported that the photocatalytic ozonation process produces a higher rate of ^•^OH radical than the other oxidation processes due to the more electrophilic nature of ozone (O_3_) than oxygen (O_2_) towards photo-generated electrons. Hence, the faster and higher rate of colour degradation and the ultimate decomposition of organics and mineralization is possible by the photocatalytic ozonation process [13]. Therefore, the modified Ni-NiO/C/g-C_3_N_4_ composite could be considered as an economical and reusable catalyst in an integrated PC/O_3_ process for the complete degradation of organic dyes, including CR and ARS, due to rapid and high degradation with the lowest energy consumption.

### 3.4. Treatment of Real Industrial Wastewater

Generally, the wastewater that comes from the food and juice industries is coloured and smells, as well as having a high level of chemical oxygen demand (COD), biological oxygen demand (BOD), suspended solids (SS), etc. [56,57]. The effluents from the food and juice industries may contain sugar, nutrients with added minerals and colours, preservatives, and additives, which may cause fast microbial growth, poor visibility, and foul odour [58]. The chemical oxygen demand (COD) is a vital indicator of the presence of organic pollutants, including dyes, in the wastewater [59], so the concentration of COD and degradation of COD will be the parameters of the removal of pollutants in wastewater. This study collected wastewater from the juice factory in Jeddah, Saudi Arabia. After the primary analysis of fruit wastewater, as seen in Table 2, the subsequent experiments, such as photocatalysis, ozonation, and integrated PC/O_3_, were conducted to evaluate and compare the efficiencies of all three methods studied for COD removal from industrial wastewater.

**Figure 9:**
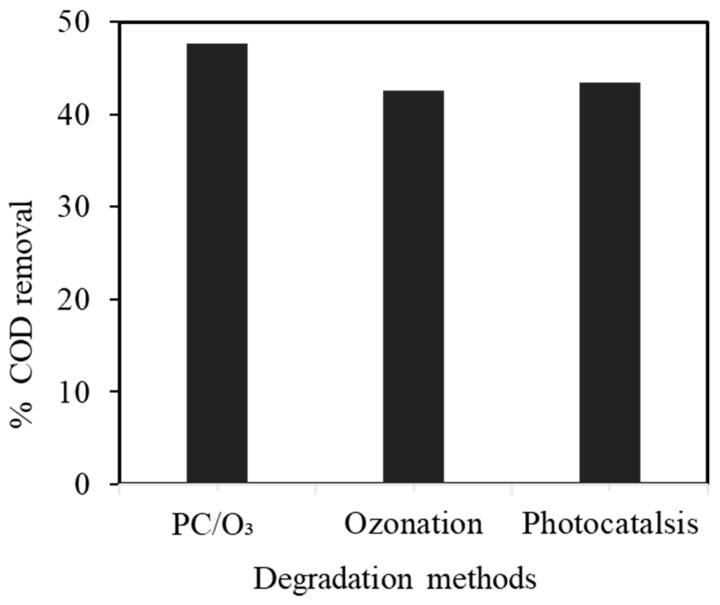
COD removal by photocatalysis, ozonation, and integrated photocatalytic ozonation (initial COD concentration 4200 mg/L, pH 4.0, Vol. 50 mL, contact time 180 min, catalyst: Ni-NiO/C/g-C_3_N_4_, catalyst mass 0.05 g, ozone flow rate 20 g/h).

The results in Figure 9 show that the best efficiency was in the integrated PC/O_3_ process, followed by ozonation and photocatalytic degradation at 47.61%, 42.59%, and 34.4%. This may be due to the synergistic effect of highly oxidative photoelectrons by ozonation, producing a high rate of ^•^OH radicals and triggering the semiconducting modified Ni-NiO/C/g-C_3_N_4_ composite for the effective mineralization of organic pollutants.

### 3.5. Catalyst Stability Study

**Figure 10:**
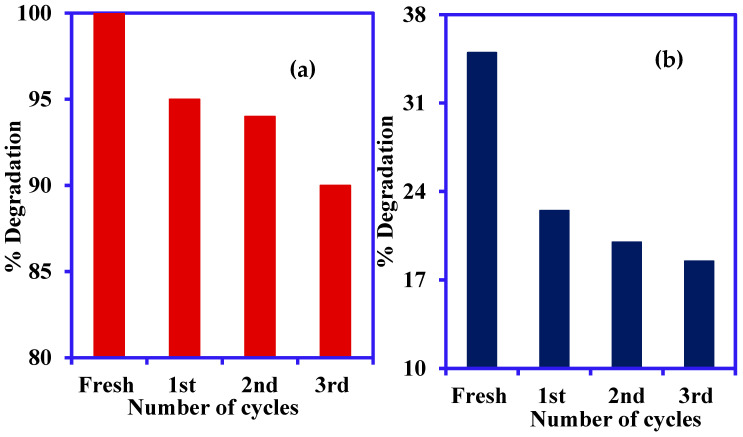
Regeneration study of (**a**) CR and (**b**) ARS degradation using the Ni-NiO/C/g-C_3_N_4_ composite (concentration 20 mg/L, pH 5.0 for CR and 9.0 for ARS, contact time 180 min, Vol. 50 mL, catalyst: Ni-NiO/C/g-C_3_N_4,_ catalyst mass 0.05 g).

The regeneration of catalysts is one of the key aspects of the commercial use and stability of photocatalysts. Under optimum operating conditions, the Ni-NiO/C/g-C_3_N_4_ composite reusability for CR and ARS degradation was assessed. Before reusing, the Ni-NiO/C/g-C_3_N_4_ composite was cleaned with deionized water and dried at 80 °C. In Figure 10, experimental findings demonstrate that the Ni-NiO/C/g-C_3_N_4_ composite continues to function well after being reused four times for CR and ARS degradation, respectively. As the number of cycles increased, the NiO/C/g-C_3_N_4_ composite effectiveness in CR and ARS degradation was slightly reduced. These findings imply that the Ni-NiO/C/g-C_3_N_4_ is robust and suitable for repeated use.

### 3.6. Degradation Mechanisms of CR and ARS

**Figure 11:**
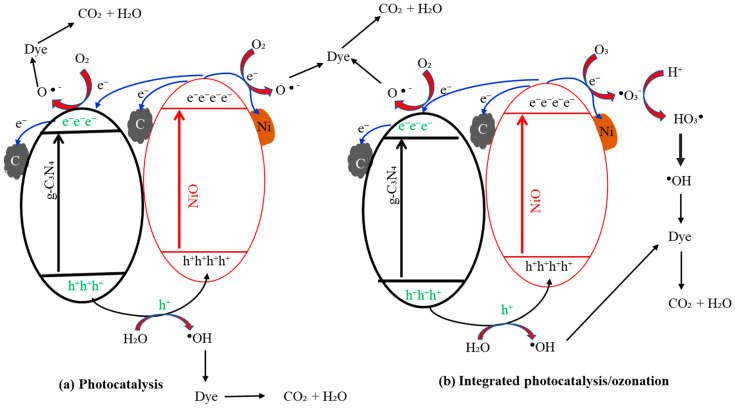
A schematic diagram showing the production of active radical species under (**a**) photocatalysis and (**b**) integrated photocatalysis–ozonation.

The degradation mechanisms of CR and ARS using Ni-NiO/C/g-C_3_N_4_ were mainly proposed by two pathways, including reduction and oxidation processes, as shown in Figure 11a. The photo-generated electron/hole pairs are produced by the Ni-NiO/C/g-C_3_N_4_ catalyst under visible light illumination. Upon energy absorption, the semiconductor catalyst’s valence band (VB) electrons were stimulated to the conduction band (CB), leaving a positively charged hole at the VB. The electron–hole pair arises as a result of this [38]. Superoxide radicals (^•^O_2_^−^) are free radicals created when the significant reduction potential of the electron in the CB reduces O_2_ in a solution. Due to their high oxidizing potential, the holes left in the VB might oxidize the H_2_O molecules and create ^•^OH [60]. The CR and ARS molecules were broken down into CO_2_ and H_2_O molecules by the free radicals that were produced [61]. The possible separation of photo-induced electron–hole pairs in the Ni-NiO/C/g-C_3_N_4_ catalyst and the decomposition of the dye moles are demonstrated in Figure 11a.

The CR and ARS degradation by ozone in aqueous solution is mainly by direct and indirect pathways. Firstly, it might be the direct oxidation of CR and ARS by molecular ozone, which involves selective reactions, such as electrophilic, nucleophilic, or dipolar addition reactions with low reaction rates [62]. At the same time, another CR and ARS removal mechanism was proposed, and the indirect reaction is the decomposition of ozone to produce ^•^OH radicals that are non-selective and extremely reactive with CR and ARS [63]. Both ozone and ^•^OH radicals were involved as strong chemical oxidants for the mineralization of CR and ARS from the solution [64].

The mechanisms were proposed in the integrated PC/O_3_ process for the enhanced degradation of CR and ARS. Since the redox potential of oxygen and oxygen peroxide in the reaction medium is higher than the CB potential of the Ni-NiO/C/g-C_3_N_4_, electrons move to the oxygen in the reaction medium. They are abundantly transformed into oxygen [64]. The use of photo-generated carriers and the formation of reactive oxygen species (ROS) will impact the photocatalytic system [49]. When injected into photocatalytic systems, electrophilic ozone readily captures photo-generated electrons in contrast to its direct ozonation [49]. The electron–hole pairs are effectively separated under simulated light irradiation, resulting in the efficient transfer of the photo-generated electrons of Ni-NiO/C/g-C_3_N_4_, which causes a significant amount of ROS to be formed [65]. The ozone consumption ratio of the O_3_/light system can also be increased using a Ni-NiO/C/g-C_3_N_4_ catalyst. Abundant ozone molecules increased the reaction’s production of ROS, which helped the mineralization of CR and ARS in an integrated system. A detailed mechanism for the decomposition of dye molecules under an integrated PC/O_3_ process is shown in Figure 11b.

## 4. Conclusions

In this study, the Ni-NiO/C/g-C_3_N_4_ composite catalyst was fabricated by applying a hydrothermal method and subsequently applied for the degradation of CR and ARS from wastewater in an integrated PC/O_3_. The catalysts were characterized using SEM, XRD, XPS, and UV-Vis DRS. The morphological features, physical appearance, state of chemical elements, and optical behaviour of catalysts confirmed the successful fabrication of the catalysts. The factors affecting the photocatalytic ozonation were evaluated, including contact time, solution pH, initial dye concentration, etc. The degradation rate of CR and ARS was compared between separate photocatalysis, ozonation, and integrated PC/O_3_ methods with a remaining constant of operating factors such as pH 5.0 for CR and pH 9.0 for ARS, an initial concentration of 20 mg/L for both dyes, a catalyst mass of 0.05 g for 50 mL of each sample solution under visible light illumination, and an ozone rate of 20 g/h. The results showed the complete (100%) degradation of CR and ARS within 5 min and 40 min of reaction time, respectively, by integrated PC/O_3_ in the presence of a modified Ni-NiO/C/g-C_3_N_4_ composite catalyst. The reusability of the modified catalyst was evaluated, and four successive regenerations were achieved. The modified Ni-NiO/C/g-C_3_N_4_ composite could be considered an economical and reusable catalyst in an integrated PC/O_3_ process for the complete degradation of organic dyes, including CR and ARS, due to the rapid and high degradation with the lowest energy consumption.

## Figures and Tables

**Table 1:** Degradation of CR and ARS using photocatalytic, ozonation, and integrated photocatalytic ozonation methods.

Degradation Method	Pollutant and (% Efficiency)	Conditions						Refs.
		Catalyst	pH	Conc. (mg/L)	* LS	** CT (min)	*** OR	
Photocatalysis	CR (99.5%)	Ag_2_WO_4_/Ag_2_S	-	20	Vis	-	-	[51]
Photocatalysis	CR (98%)	Ag_2_WO_4_/Bi_2_S_3_	-		Vis	60	-	[52]
Photocatalysis	ARS (92%)	MgO@ZrO_2_@g-C_3_N_4_	7.0	10	Vis	60	-	[53]
Photocatalysis	CR (100%) ARS (40%)	NiO/C/g-C_3_N_4_	5.0 9.0	20	Vis	180	-	This study
Ozonation	CR (85%)	-	10.0	25	-	60	36 mg/h	[54]
Ozonation	ARS (40%)	-	9.0	100	-	10	5 g/h	[9]
Ozonation	CR (100%) ARS (100%)	-	5.0 9.0	20	-	120	20 g/h	This study
Photocatalytic ozonation	ARS (40%)	PAC/γ-Fe_2_O_3_/O_3_	9.0	100	UV	10	5 g/h	[9]
Photocatalytic ozonation	ARS (92%)	Copper-doped zinc oxide/Ozon (ZCO)/O_3_	-	-	UV-Vis	160	-	[55]
Photocatalytic ozonation	CR (100%) ARS (100%)	NiO/C/g-C_3_N_4_/O_3_	5.0 9.0	20	Vis	5 and 40	20 g/h	This study

* LS = Light source; ** Contact time; *** Ozone rate.

**Table 2:** The characteristics of wastewater from the Al-Saham juice factory in Jeddah, Saudi Arabia.

S. No.	Parameter	Unit	Results	Standard Limit (* MEWA, KSA)
1	pH	pH	4	5–10
2	Total dissolved solids (TDS)	mg/L	172	3000
3	Dissolved oxygen (DO)	mg/L	0.3	NA **
4	Total solid (TS)	mg/L	3	NA
5	Total volatile solid (TVS)	mg/L	0.16	NA
6	Total suspended solid (TSS)	mg/L	0.05	600
7	Chemical oxygen demand (COD)	mg/L	4700	1000
8	Total organic carbon (TOC)	mg/L	577	NA

* Ministry of Environment, Water, and Agriculture, Kingdom of Saudi Arabia. ** NA = Not Available.

## Data Availability

Data are contained within the article.
